# Effects of Methyl Jasmonate on Fruit Quality and Disease Resistance of Mechanically Injured Banana Fruit During Storage

**DOI:** 10.1002/fsn3.71392

**Published:** 2025-12-28

**Authors:** Fang Huang, Zhenmei Fan, Ping Yi, Min Huang, Ting Gan, Yuhan Long, Lihong Xie, Li Li, Lan Zhang, Hao Dong, Qi Wei

**Affiliations:** ^1^ Agro‐Food Science and Technology Research Institute Guangxi Academy of Agricultural Sciences Nanning China; ^2^ Guangxi University Nanning China; ^3^ Guangxi Key Laboratory of Fruits and Vegetables Storage‐Processing Technology Nanning China

**Keywords:** banana, fruit quality, mechanical injury, methyl jasmonate

## Abstract

Mechanical damage to banana fruit during transportation leads to substantial quality loss and shortened shelf life. This study investigated the effects of different concentrations (10, 20, and 50 μM) of methyl jasmonate (MeJA) on mechanically injured banana fruits stored at 25°C for 14 days. The results showed that the 10 μM MeJA treatment was identified as optimal, delaying the respiration peak by 2 days and maintaining fruit greater firmness (95.86 N) on Day 14. It also effectively suppressed the rise in ripening index, decay rate, total soluble solids (TSS) content, and malondialdehyde (MDA) content, thereby preserving better appearance and edible quality. In addition, 10 μM MeJA enhanced antioxidant enzyme activities and strengthened the activities of enzymes associated with disease resistance. On Day 14, the activities of ascorbate peroxidase (APX) and phenylalanine ammonia‐lyase (PAL) were significantly higher than those of the control by 2.02‐ and 1.73‐fold, respectively. Meanwhile, the activities of peroxidase (POD), polyphenol oxidase (PPO), β‐1,3‐glucanase (GLU), and chitinase (CHT) were maintained at levels 48.37%, 37.30%, 13.61%, and 47.77% higher than the control, respectively, at the end of storage. This study indicated that treatment with 10 μM MeJA effectively enhanced the antioxidant ability and disease resistance of mechanically injured banana fruits, thereby maintaining fruit quality during storage and improving the storability.

## Graphical abstract

Treatment with MeJA delays ripening and reduces membrane lipid peroxidation of mechanically injured banana fruit. Treatment with MeJA enhances antioxidant ability and disease resistance of mechanically injured banana fruit. 10 μM MeJA effectively maintains fruit quality of mechanically injured banana fruit than that of 20 and 50 μM MeJA.

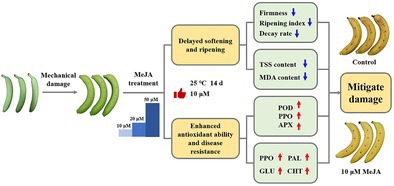

## 1 Introduction

Banana ( *Musa acuminata* L.), an important tropical and subtropical fruit, is the most widely consumed fruit worldwide (Huang et al. 2014; Li et al. 2017a). Owing to their aromatic odor, unique sweet flavor, high nutritional value, and abundance of essential micronutrients and vitamins required by the human body, banana fruits are favored by consumers (Ranjha et al. 2022). However, the banana fruits are susceptible to mechanical injury during storage and transportation, leading to quality deterioration and spoilage after harvest, which affects the shelf life and market value (Li et al. 2017b). Mechanical damage primarily includes injuries to fruits and vegetables caused by compression, bruising, impact, vibration, abrasion, piercing, and cutting during harvesting, packaging, transportation, sales, and processing (Al‐Dairi et al. 2023). Suffering mechanical damage, the respiration rate and ethylene production rate of bananas increase, resulting in tissue softening, increased disease susceptibility, and rapid senescence and spoilage (Maia et al. 2011; Pathare and Al‐Dairi 2022; Al‐Dairi et al. 2024).

Jasmonate (MeJA) is an endogenous signaling molecule and natural compound in plants, which has attracted significant attention for its direct application in fruit storage and preservation (Khaksar et al. 2017). It has been shown to enhance the resistance of citrus (Liao, Li, et al. 2022), mango (Huang, Liu, et al. 2024), peach (Zhu et al. 2022), and avocado (Glowacz et al. 2017). Previous studies have shown that MeJA primarily triggers plant disease resistance responses by activating key components of the jasmonic acid signaling pathway (MYC2, JAZ proteins) (Ali and Baek 2020; Liao, Pan, et al. 2022). On one hand, MeJA mainly regulates enzymes (SOD, POD) related to reactive oxygen species metabolism, reducing the accumulation of free radicals and alleviating membrane lipid peroxidation damage (Pan et al. 2020). On the other hand, MeJA modulates the activity and gene expression of enzymes (PLA, PPO, 4CL) involved in the phenylpropanoid metabolic pathway, thereby increasing the accumulation of lignin and phenolic compounds and strengthening the cell wall structure to inhibit pathogen invasion (Ji, Wang, Li, et al. 2021; Yuan et al. 2024). Additionally, MeJA can maintain the energy supply required for disease resistance (Zhao et al. 2022) and increase the accumulation of pathogenesis‐related proteins to enhance disease resistance (Ji, Wang, Zuo, et al. 2021). Gu et al. (2022) demonstrated that MeJA could repair the mechanical damage of sweet cherries by enhancing the antioxidant capacity and the phenylpropane metabolism. Xu et al. (2020) revealed that MeJA inhibited senescence and floret yellowing of broccoli by regulating the antioxidant system and chlorophyll metabolism. Similar results were observed in mechanically wounded cucumber (Peng and Mao 2011) and fresh‐cut Chinese water chestnuts (Lu et al. 2024). These findings indicated that MeJA treatment could serve as an effective strategy to mitigate mechanical injuries in postharvest horticultural crops.

Our previous research found that puncture injury could induce an increase in phospholipase D (PLD) activity in bananas (Li et al. 2017a, 2017b), and treatment with PLD inhibitor could alleviate fruit damage stress (Li et al. 2022). Meanwhile, MeJA also alleviated banana puncture injury by regulating membrane lipid metabolism (Huang, Yi, et al. 2024; Huang, Liu, et al. 2024). However, the effects of MeJA treatment on the quality maintenance, antioxidant capacity, and disease resistance of puncture‐injured bananas remain undetermined. Thus, in this study, we have investigated the changes in quality parameters, antioxidant, and disease‐resistant enzyme activities of mechanically damaged fruits, and determined the optimal MeJA concentration for maintaining fruit quality, antioxidant capacity, and disease resistance. This study aims to provide a theoretical basis for further research into the mechanism by which MeJA regulates mechanically injured bananas, as well as its application in banana preservation.

## 2 Materials and Methods

### 2.1 Fruit Materials and Treatments

Banana fruits were harvested from an orchard in Tanluo Town, Nanning City, Guangxi Province, China. The banana fruits of uniform size, without mechanical injury, diseases, or insect pests, were selected at the mature green stage and transported to the laboratory within 4 h of harvest. The selected bananas were sterilized with a 0.1% sodium hypochlorite solution for 10 min, rinsed with sterile distilled water, and subsequently air‐dried.

All fruits were mechanically wounded in 1‐mm‐deep injuries using a 2 mm‐diameter stainless‐steel needle as described by Li et al. (2017b). The injured banana fruits were randomly divided into four groups (*n* = 200 per group), which were immersed in different solutions: distilled water (as control group, CK), 10, 20, and 50 μM MeJA solution (as experimental groups), respectively. Next, the treated fruits were air‐dried and packaged in polyethylene bags (thickness of 0.03 mm) and stored at 25°C, with a relative humidity of 90%–95% for 14 days. Three replicates from each group were analyzed on days 0, 2, 4, 6, 8, 10, 12, and 14 during storage.

### 2.2 Determination of the Ripening Index and Decay Rate

The ripening index was evaluated using a color scale (0–6) according to the approach of Zhu et al. (2020) with minor modifications. Specifically, 0 = entirely green, 1 = beginning to break green, 2 = predominantly green with some yellow, 3 = predominantly yellow with some green, 4 = yellow with green tips, 5 = entirely yellow, and 6 = entirely yellow with visible brown freckles. Twenty fixed banana fruits were selected for each treatment, and the relevant parameters were measured every 2 days. The ripening index was then calculated according to the following equation:

(1) $$\text{Ripening index}\left(\%\right)=\frac{\sum \left(Y_{i}\times N_{i}\right)}{7\times N_{0}}\times 100\%$$

where *Y*_*i*_ is the yellowing scale, *N*_*i*_ is the number of fruits at that yellowing scale, and *N*_0_ is the total number of banana fruits.

The decay rate of banana fruits was assessed using the modified approach of Xu et al. (2019). Twenty fixed bananas of each treatment were selected to measure the decay rate, which was calculated using the following formula:

(2) $$\text{Decay rate}\left(\%\right)=\frac{\text{Number of rotten fruits}}{\text{Total number of stored fruits}}\times 100\%$$

### 2.3 Determination of Respiratory Rate, Firmness, and Total Soluble Solids Content

To measure the respiratory rate of banana fruits, a modified method of Li et al. (2022) was utilized. Three fruits were enclosed within a 4.2‐L glass jar (one jar per replicate) at 25°C. After 2 h, 1 mL of headspace gas was extracted and assessed using a Shimadzu GC‐9A gas chromatograph (Kyoto, Japan) with a Poropak N column and a thermal conductivity detector. The concentration of carbon dioxide was recorded, and the respiratory rate was represented on a fresh weight basis.

The firmness of banana fruit was measured utilizing a GY‐4 fruit hardness tester (Zhejiang TOP Instrument). Three fruits were randomly selected from each banana, peeled, and cut in half to measure two points on the cut surface. The results are expressed as the average of six measurements in units of Newtons (N).

Total soluble solids (TSS) content in banana pulp was measured using a handheld refractometer (Fisher Scientific, Canada) following AOAC protocols. Six replicate measurements were performed, and the mean value was calculated as the final TSS content.

### 2.4 Measurement of the Malondialdehyde Content

Malondialdehyde (MDA) content was determined following the protocol described by Li et al. (2017b). 0.25 g frozen peel sample was mixed with 0.1% trichloroacetic acid (TCA) and centrifuged at 15,000 *g* for 15 min. The supernatant was added to 3 mL of 0.5% 2‐thiobarbituric acid (TBA) solution, boiled for 20 min, and cooled immediately in ice. Finally, the absorbance of the mixture was measured at 450, 532, and 600 nm. MDA content was calculated as millimoles per gram of fresh weight (mmol g^−1^).

### 2.5 Measurement of Enzyme Activities

Peroxidase (POD) activity was determined using POD Activity Assay Kit (Solarbio BC0090, Beijing, China). Catalase (CAT) activity was measured using CAT Activity Assay Kit (Solarbio BC0200). Ascorbate peroxidase (APX) activity was measured using APX Activity Assay Kit (Solarbio BC0220). Polyphenol oxidase (PPO) activity was determined using PPO Activity Assay Kit (Solarbio BC0190). Phenylalanine ammonia‐lyase (PAL) activity was assessed using PAL Activity Assay Kit (Solarbio BC0210). Furthermore, β‐1,3‐glucanase (GLU) activity was measured using GLU Activity Assay Kit (Solarbio BC0360). Chitinase (CHT) activity was determined using CHT Activity Assay Kit (Solarbio BC0820). All enzyme activities were measured as manufacturer's protocol and the results were expressed as U/g.

### 2.6 Statistical Analysis

All experiments were conducted in triplicate (*n* = 3) utilizing a fully randomized design. The data were expressed as the mean ± standard error (SE) and analyzed through SPSS software (version 26.0). Duncan's multiple range test was employed to assess the significance of differences among the data.

## 3 Results

### 3.1 Ripening Index and Decay Rate

**FIGURE 1:**
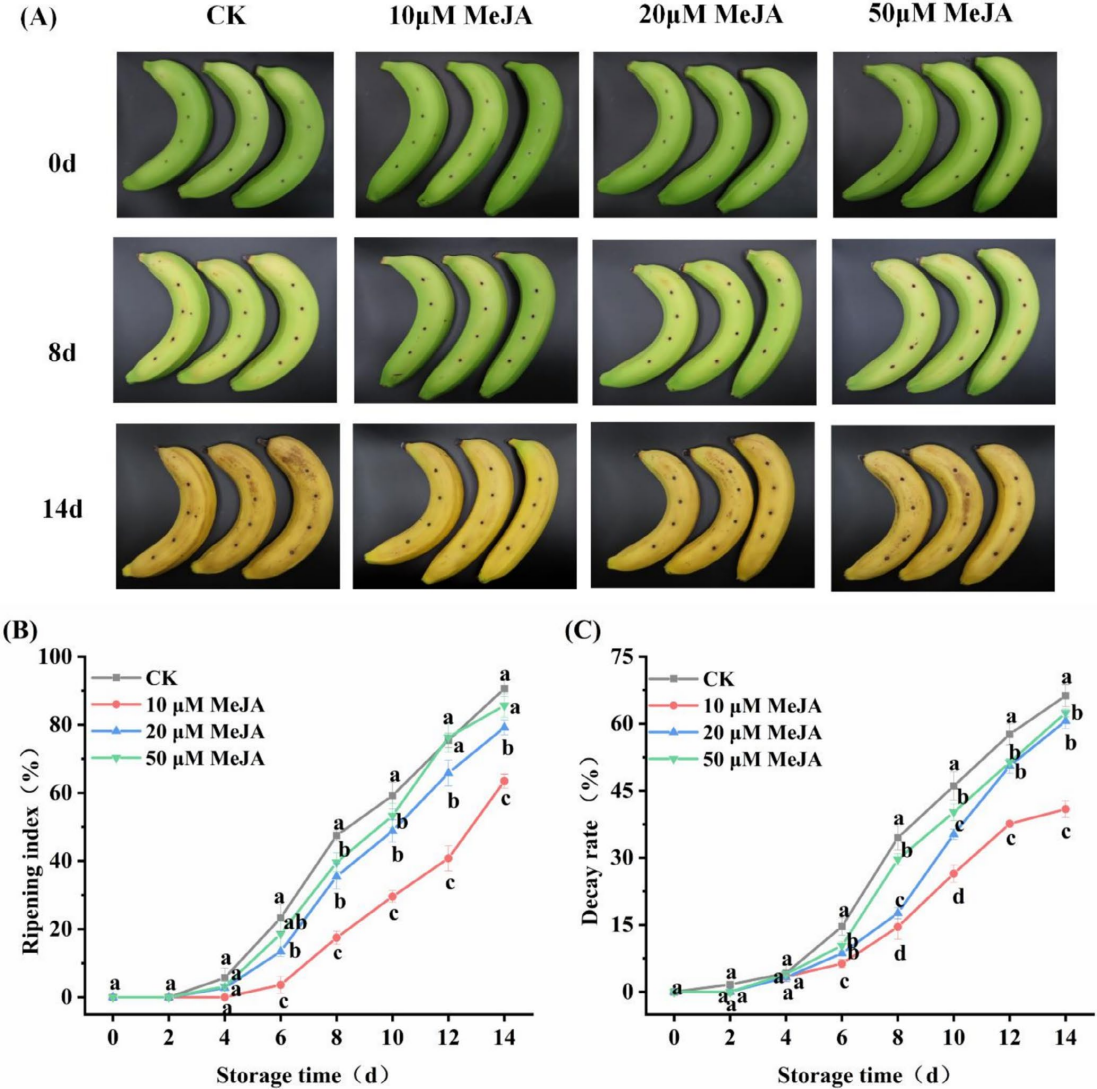
Effect of MeJA treatment on apparent indicators of banana, (A) appearance, (B) ripening index, (C) decay rate. The data represent mean ± SD (*n* = 3). Significant differences (*p* < 0.05) among different treatments on the same storage day were indicated by different lowercase letters.

During the fruit ripening and senescence process, the color of banana peel gradually changed from green to yellow (Figure 1A). As shown in Figure 1B, the ripening index of the banana peel increased progressively with extended storage time. After 14 days of storage, 10 and 20 μM MeJA treatments effectively attenuated this increase, reducing ripening index by 29.98% and 12.57% compared to the CK, respectively (*p* < 0.05). Meanwhile, the ripening index of 10 μM MeJA treatment group increased most slowly. It was significantly lower than that of the CK by 63.17%, 50.05%, 45.93%, and 29.98% on days 8, 10, 12, and 14, respectively (*p* < 0.05). These results demonstrate that 10 μM MeJA treatment effectively postponed the yellowing of the banana peel.

As shown in Figure 1C, with prolonged storage period, the decay rate of bananas gradually increased. Compared with CK, MeJA treatment, at three concentrations, effectively delayed the increase in the banana decay rate during the storage duration. On Day 14, the decay rates of 20 and 50 μM MeJA treatment groups were lower than those of CK by 9.11% and 5.77%, respectively (*p* < 0.05). Particularly, 10 μM MeJA treatment yielded the most pronounced effect, showing a highly significant reduction of 38.30% (*p* < 0.05). These findings suggest that treatment with 10 μM MeJA effectively mitigates postharvest decay in mechanically damaged bananas, consequently prolonging their storage period.

### 3.2 Respiratory Rate, Firmness, TSS, and MDA Content

**FIGURE 2:**
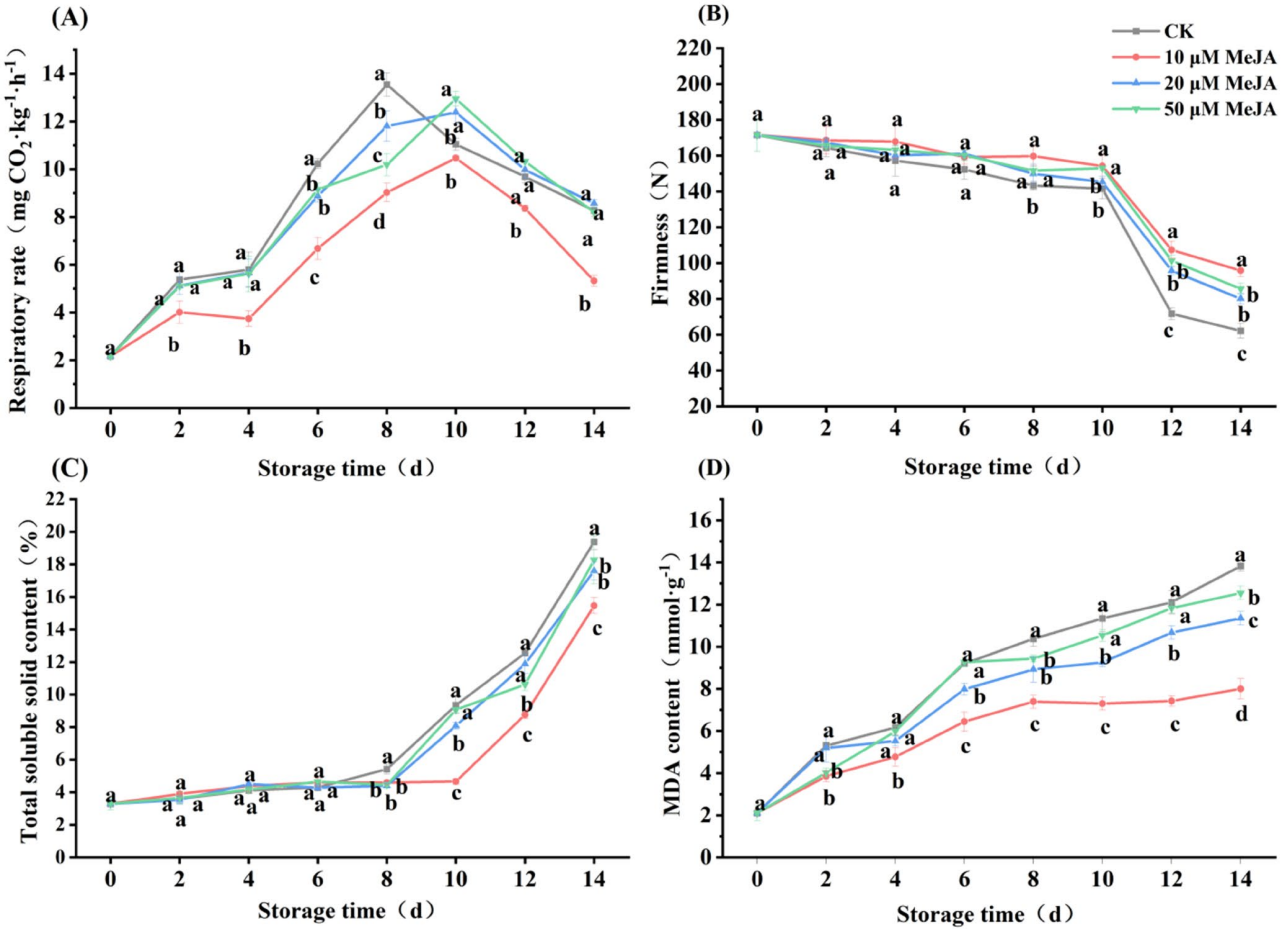
Effect of MeJA treatment on (A) the respiratory rate, (B) firmness, (C) total soluble solids content, (D) MDA content of banana. The data represent mean ± SD (*n* = 3). Significant differences (*p* < 0.05) among different treatments on the same storage day were indicated by different lowercase letters.

In Figure 2A, the respiratory rate exhibited an overall trend of initially increased and then declined during storage. The respiratory peak in the CK occurred on Day 8 at 13.54 mg CO_2_ kg^−1^ h^−1^, whereas all MeJA‐treated groups peaked 2 days later on Day 10. Furthermore, the 10 μM MeJA group not only showed the lowest peak (10.47 CO_2_ kg^−1^ h^−1^) but also maintained a respiration rate significantly lower than the CK at all time points (*p* < 0.05), effectively delaying the injury‐induced respiratory rise.

As shown in Figure 2B, the fruit firmness declined gradually during the early storage period, followed by a sharp decrease on Day 12. At the end of storage, the firmness of the 10, 20, and 50 μM MeJA treatments was significantly higher than that of the CK by 54.32%, 19.65%, and 11.92%, respectively (*p* < 0.05). And 10 μM MeJA of firmness maintained a high value was 95.86 N on Day 14. Overall, the 10 μM MeJA treatment was most effective in alleviating fruit softening.

As presented in Figure 2C, TSS increased continuously throughout storage. In the initial 8 days, the rise in TSS was comparatively gradual, with no remarkable difference (*p* > 0.05) between the CK and MeJA treatments. However, by Day 14, TSS contents of the 10, 20, and 50 μM MeJA groups were found to be 20.21%, 9.20%, and 5.76% significantly lower than those in the CK group, respectively (*p* < 0.05). The 10 μM MeJA group consistently maintained the lowest TSS content from Day 10 onwards, indicating that it effectively delayed starch‐to‐sugar conversion and slowed TSS accumulation, thereby retarding ripening of bananas.

As shown in Figure 2D, the MDA content increased with the extension of storage, and MeJA treatments inhibited the accumulation of MDA content compared to the CK. Specifically, on Day 14, the treatments with 10, 20, and 50 μM MeJA resulted in significant reductions of 42.14%, 17.92%, and 9.30% when compared to the CK (*p* < 0.05). Furthermore, the MDA content in the 10 μM MeJA group was significantly lower than that of the CK group throughout the entire storage period. Therefore, 10 μM MeJA has the greatest potential in suppressing the accumulation of MDA in mechanically injured bananas.

### 3.3 The Effect of MeJA on POD, CAT, and APX Activities

**FIGURE 3:**
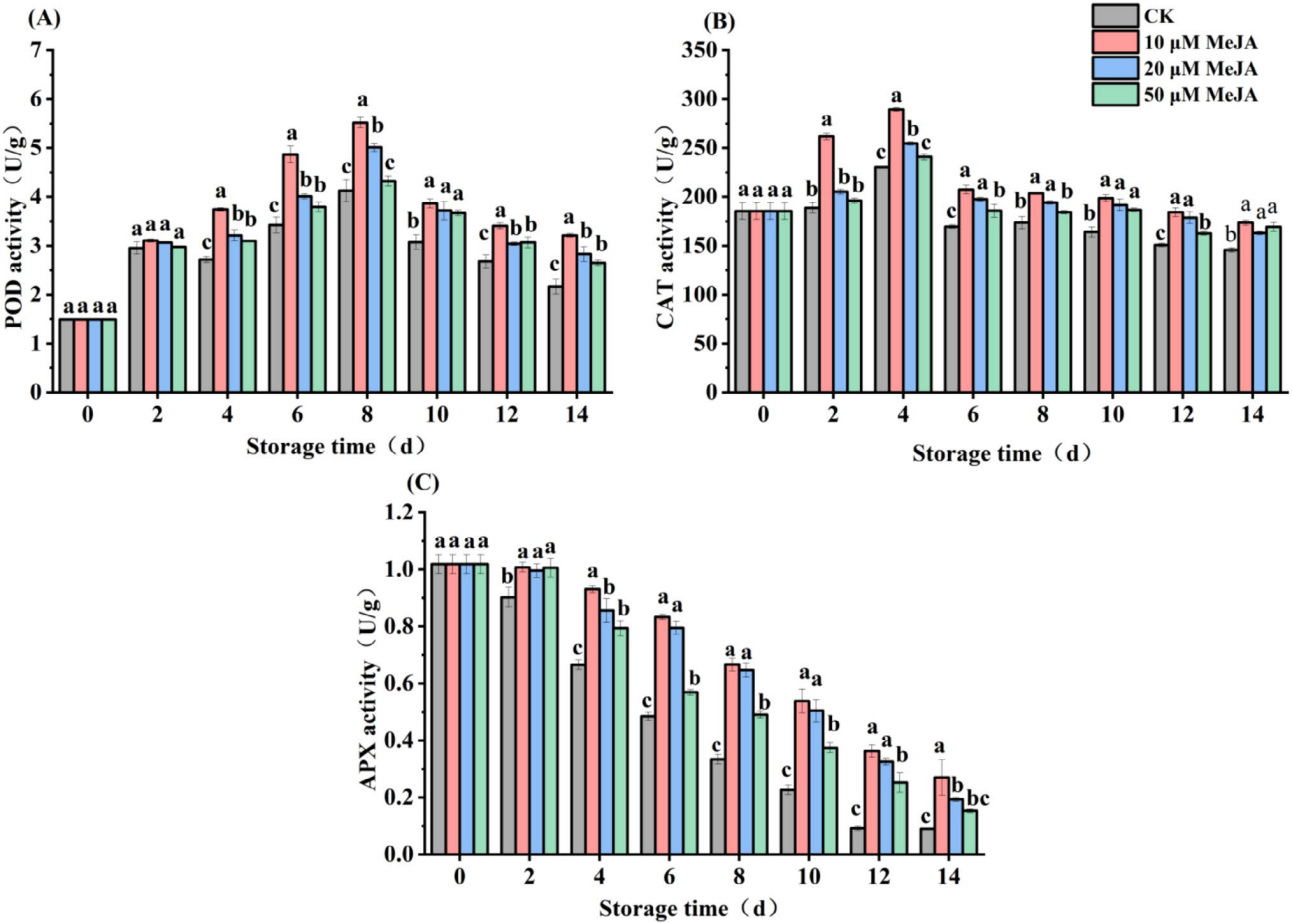
Effect of MeJA treatment on (A) peroxidase (POD), (B) catalase (CAT), (C) ascorbate peroxidase (APX) activities of banana. The data represent mean ± SD (*n* = 3). Significant differences (*p* < 0.05) among different treatments on the same storage day were indicated by different lowercase letters.

Figure 3A illustrated that POD activity in mechanically injured bananas initially increased, followed by a subsequent decline. On Day 8 of storage, all groups reached their peak POD activity; 10 and 20 μM MeJA treatments significantly (*p* < 0.05) enhanced POD activity by 33.78% and 21.49%, respectively, compared to the CK, whereas 50 μM MeJA treatment did not show a significant effect (*p* > 0.05). In addition, the 10 μM MeJA group consistently maintained the highest POD activity from Day 4 onwards, and its activity was 48.37% higher than that of CK on Day 14. This indicated that treatment with 10 μM MeJA can significantly enhance POD activity and sustain relatively elevated activity levels during the entire storage time.

As shown in Figure 3B, with a prolonged storage period, CAT activity exhibited a trend of first increasing and then decreasing. Bananas treated with 10 μM MeJA showed a rapid and significant (*p* < 0.05) increase in CAT activity on Day 2 of storage by 38.57% compared with the CK. On Day 4 of storage, CAT activity reached its peak across all groups, with the 10 μM MeJA treatment group maintaining a high level (289.27 U/g) and exhibiting a significant increase (*p* < 0.05) of 25.51% relative to CK. From Day 6 to Day 14, the activities of CAT in three concentrations of MeJA treatment groups were notably higher (*p* < 0.05) than those of CK, indicating that MeJA enhanced CAT activity in mechanically injured bananas.

Figure 3C shows the APX activity gradually decreased with prolonged storage period. Throughout the storage period, APX activities in 10 and 20 μM MeJA bananas remained significantly (*p* < 0.05) higher than those in CK. At the end of the storage, the APX activities of 10 and 20 μM MeJA were 2.02 and 1.16 times higher than those of the CK, respectively. Therefore, treatments with 10 and 20 μM MeJA effectively delayed the decline in APX activity of bananas.

### 3.4 The Effect of MeJA on PPO and PAL Activities

**FIGURE 4:**
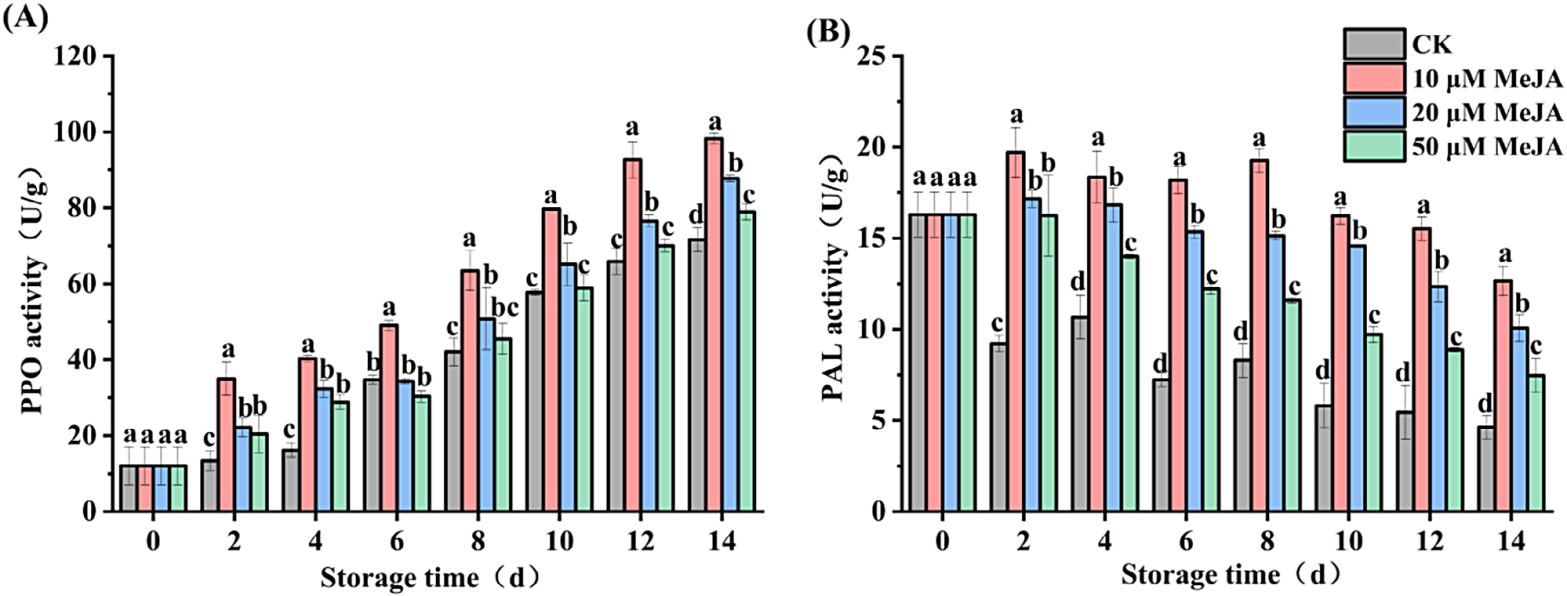
Effect of MeJA treatment on (A) polyphenol oxidase (PPO) and (B) phenylalanine ammonia‐lyase (PAL) activities of banana. The data represent mean ± SD (*n* = 3). Significant differences (*p* < 0.05) among different treatments on the same storage day were indicated by different lowercase letters.

There was an upward trend in PPO activity during the entire storage duration (Figure 4A). On Day 14, the 10 μM MeJA group had the highest PPO enzyme activity (98.26 U/g) among all groups, and its activity was significantly higher than the CK by 37.30%. 10 μM MeJA also maintained elevated PPO activity throughout the storage. Therefore, treatment with 10 μM MeJA significantly enhanced PPO activity in bananas.

Compared with CK, treatment with 10 μM MeJA significantly enhanced PAL activity in bananas throughout the storage period (Figure 4B). Collectively, the MeJA across treatment concentrations differentially enhanced PAL activity, with the most substantial enhancement observed in 10 μM MeJA. Treatment with 10 μM MeJA resulted in a peak PAL activity of 19.71 U/g on Day 2 and a level 1.73 times higher than the CK on Day 14. These results suggest that 10 μM MeJA is the most effective in maintaining the relatively high PAL activity in bananas during storage.

### 3.5 The Effect of MeJA on GLU and CHT Activities

**FIGURE 5:**
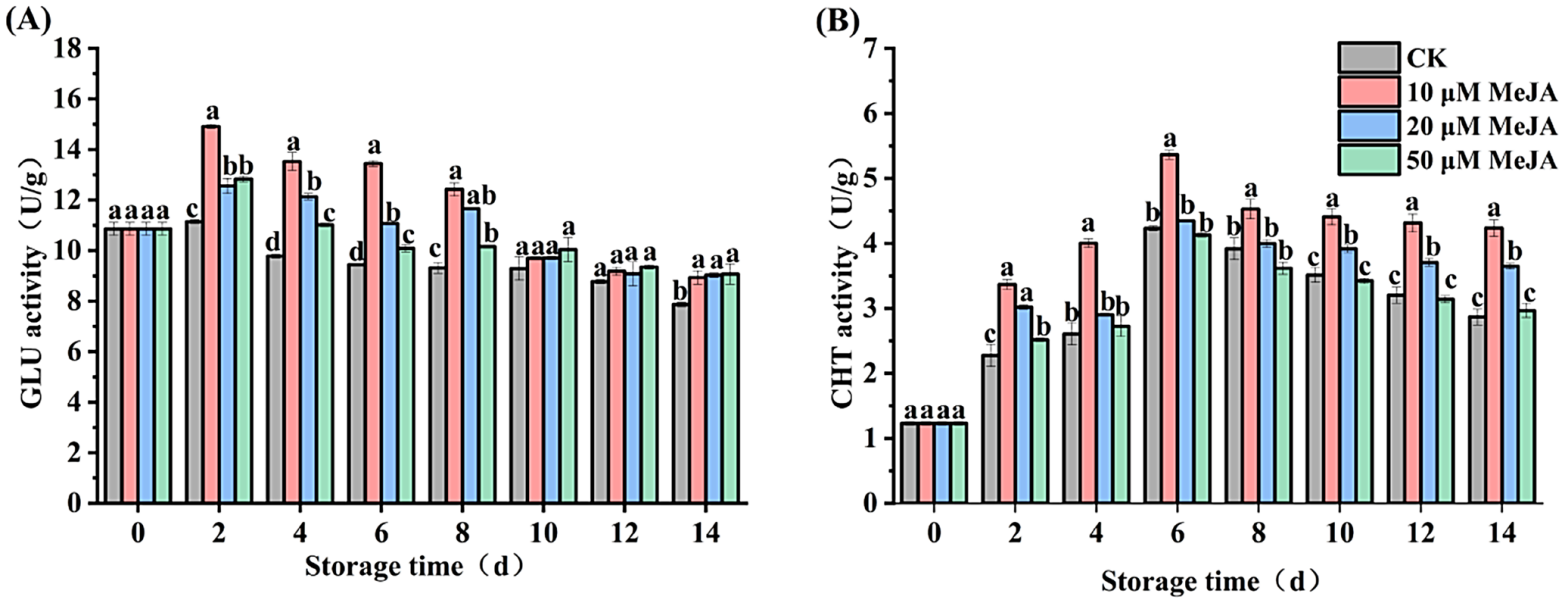
Effect of MeJA treatment on (A) β‐1,3‐glucanase (GLU) and (B) chitinase (CHT) activities of banana. The data represent mean ± SD (*n* = 3). Significant differences (*p* < 0.05) among different treatments on the same storage day were indicated by different lowercase letters.

As shown in Figure 5A, the GLU activity of bananas exhibited an overall trend of first increasing and then decreasing during storage. On Day 2, the GLU activities of the 10, 20, and 50 μM MeJA treatment groups peaked, with values significantly higher than the CK by 33.79%, 12.66%, and 15.08%, respectively (*p* < 0.05). Furthermore, the 10 μM MeJA group maintained elevated GLU activity from Day 4 to Day 8, and its activity was 13.61% higher than the CK on Day 14. This indicates that 10 μM MeJA treatment rapidly induces GLU activity during the early stage of storage to resist pathogen invasion in bananas.

Similar to the change trend in GLU activity (Figure 5B), the CHT activities of all groups reached the peaks on Day 6, with the 10 μM MeJA treatment group showing a significant increase of 26.72% compared with CK. Conversely, there is no notable difference observed in the 20 and 50 μM MeJA treatment groups compared with CK. Additionally, the 10 μM MeJA group consistently maintained the highest CHT activity from Day 2 onwards, with a value 47.77% higher than the CK on Day 14. These findings demonstrate that 10 μM MeJA effectively boosts the CHT activity of banana fruits, thereby enhancing their disease resistance.

## 4 Discussion

Banana fruits inevitably suffer from mechanical damage during harvest and transportation. This leads to the disruption of the fruit tissue structure, increased respiratory rate, accumulation of reactive oxygen species (ROS), peroxidation of membranes, pathogen invasion, and a series of physiological activities, including accelerating fruit ripening, senescence, and even rot (Li et al. 2022; Yi et al. 2022). During storage, the ripening index and decay rate of banana peels showed an increasing trend, which was an external manifestation of banana fruit ripening and senescence. In the present study, banana fruits treated with MeJA exhibited a significantly lower ripening index and decay rate compared with the control group. This may be attributed to deferred respiration and enhanced disease resistance. The respiratory rate is an important indicator of metabolic activity in banana fruit because respiratory metabolism can lead to a series of physiological changes (Yun et al. 2022; Zhou et al. 2023). We confirmed that peak respiration in the 10 μM MeJA‐treated group was delayed by 2 days. This delay may be attributed to the antagonistic effect of MeJA‐induced respiratory enzymes. This result aligns with the findings of Fan et al. (2022) in apples, where MeJA treatment delayed senescence and prolonged the shelf life of the fruit by inhibiting respiration intensity. During postharvest storage, fruits consume nutrients to sustain life. In the present study, the soluble solid content of banana fruits treated with 10 μM MeJA was significantly reduced compared with the control group. This may result from reduced physiological metabolic activity in the banana fruits of the MeJA treatment group, which slowed the conversion of starch to sugars (Shinga and Fawole 2023). Similarly, Nasrin et al. (2022) reported a decrease in the soluble solids content together with a decrease in respiration rate in cauliflower. In addition, the firmness of banana fruits treated with 10 μM MeJA was significantly increased compared with that of the control group. A previous study found that starch in banana fruit cell walls is an important factor in maintaining banana firmness. As starch is converted into sugar, the firmness of the fruits gradually decreases during storage (Chen et al. 2022). This suggests that MeJA delays the conversion of starch into sugar in banana fruits, thereby delaying the decline in firmness.

MDA produced by banana fruits during storage is a product of cell membrane lipid peroxidation that exacerbates membrane damage and reflects the extent of lipid peroxidation and fruit aging (Ding et al. 2023; Wantat et al. 2022). During the process of fruit aging, the loss of membrane integrity is closely associated with the excessive accumulation of ROS, which is caused by O_2_^−^ and H_2_O_2_ production (Yang et al. 2014). When the accumulation of free radicals reaches a certain level, oxidation and degradation of saturated fatty acids within the cell membrane occur, which results in the disruption of plant membrane structures and an increase in MDA content (Ba et al. 2022). At the end of storage, we found that the MDA content in banana fruits treated with 10 μM MeJA was significantly reduced compared with that in the control group. These results are consistent with previous studies demonstrating that MeJA can preserve the quality of fruits, likewise lemons (Liao, Pan, et al. 2022; Liao, Li, et al. 2022), mangoes (Huang, Yi, et al. 2024; Huang, Liu, et al. 2024), and peaches (Zhu et al. 2022).

Numerous studies have found that MeJA primarily regulates the activity of defense‐related enzymes to enhance stress resistance in fruits and vegetables, thereby maintaining quality (Zhu and Ma 2007; Wang et al. 2020; Zhu et al. 2022; Li et al. 2023). Due to mechanical damage, banana fruits accumulate a large amount of ROS internally, which accelerates ripening and deterioration. POD, CAT, and APX are enzymes involved in the antioxidant system that eliminate ROS from plant cells (Liu et al. 2023). Specifically, POD not only eliminates the toxicity of compounds, such as H_2_O_2_, but also catalyzes the polymerization of phenolic substances into lignin to strengthen plant cell walls and further resist pathogen invasion (Zhu et al. 2021). In the present study, POD activity in banana fruits treated with 10 μM MeJA peaked on Day 8 of storage, showing a significant increase compared with the control group. In addition, CAT and APX can eliminate H_2_O_2_ produced by fruits, catalyzing the decomposition of H_2_O_2_ into H_2_O and O_2_ to reduce cellular oxidative damage (Zhang et al. 2024). CAT activity in banana fruits treated with 10 μM MeJA peaked on Day 4 of storage, showing a significant increase compared with the control group. APX activity from Day 2 to Day 14 of storage was significantly higher than that in the CK. Similarly, Liu et al. (2023) showed that MeJA alleviates the chilling injury of okra pods by regulating ROS metabolism. These results indicate that treatment with 10 μM MeJA enhances the antioxidant capacity of banana fruits and rapidly remediates ROS that accumulates following mechanical damage. This reduces the degree of membrane peroxidation and further slows the fruit ripening and senescence processes. The effects of MeJA on increasing antioxidant enzyme activity and enhancing a fruit's intrinsic antioxidant capacity have also been demonstrated in papayas (Li et al. 2023) and cherries (Pan et al. 2022).

The stress resistance of fruits and vegetables is related to not only the antioxidant defense system but also the accumulation of some nonenzyme substances (Wang et al. 2015). Quinone substances accumulated in plant tissues exhibit high toxicity to pathogenic microorganisms. These substances may be produced through the oxidation of phenolic substances in plants, which is facilitated by PPO. The enzyme primarily involved in the biosynthesis pathway of phenolic substances is PAL. Thus, PPO and PAL can inhibit the invasion of fruit pathogens from the perspective of promoting quinone substance synthesis (Zhang et al. 2017; Yu et al. 2021). In the present study, banana fruits treated with 10 μM MeJA showed a significant increase in PPO activity from Day 2 to Day 14 of storage compared with the CK. Moreover, PAL activity was significantly increased by 1.73 times at the end of storage. The results indicate that MeJA treatment enhances the activities of PPO and PAL in banana fruits and promotes the accumulation of quinone compounds.

GLU and CHT are two enzymes related to pathogen resistance in plants. They can enhance plant disease resistance by destroying the cell walls of pathogens, which leads to cell death (Song et al. 2022). A previous study demonstrated that MeJA treatment improved the capacity of kiwifruit to establish disease resistance against soft rot induced by *Botryosphaeria dothidea* (Pan et al. 2020). In the present study, treatment with 10 μM MeJA significantly increased the activities of disease resistance‐related enzymes in banana fruits. The activities of GLU and CHT reached their peak on Day 2 and Day 6 of storage, showing significant increases of 33.79% and 26.72% compared with the CK, respectively. This is consistent with the findings of blueberries (Wang et al. 2020) and avocados (Glowacz et al. 2017) treated with MeJA; however, the mechanisms by which MeJA reduces the incidence of postharvest diseases in mechanically injured banana fruits require further exploration.

**FIGURE 6:**
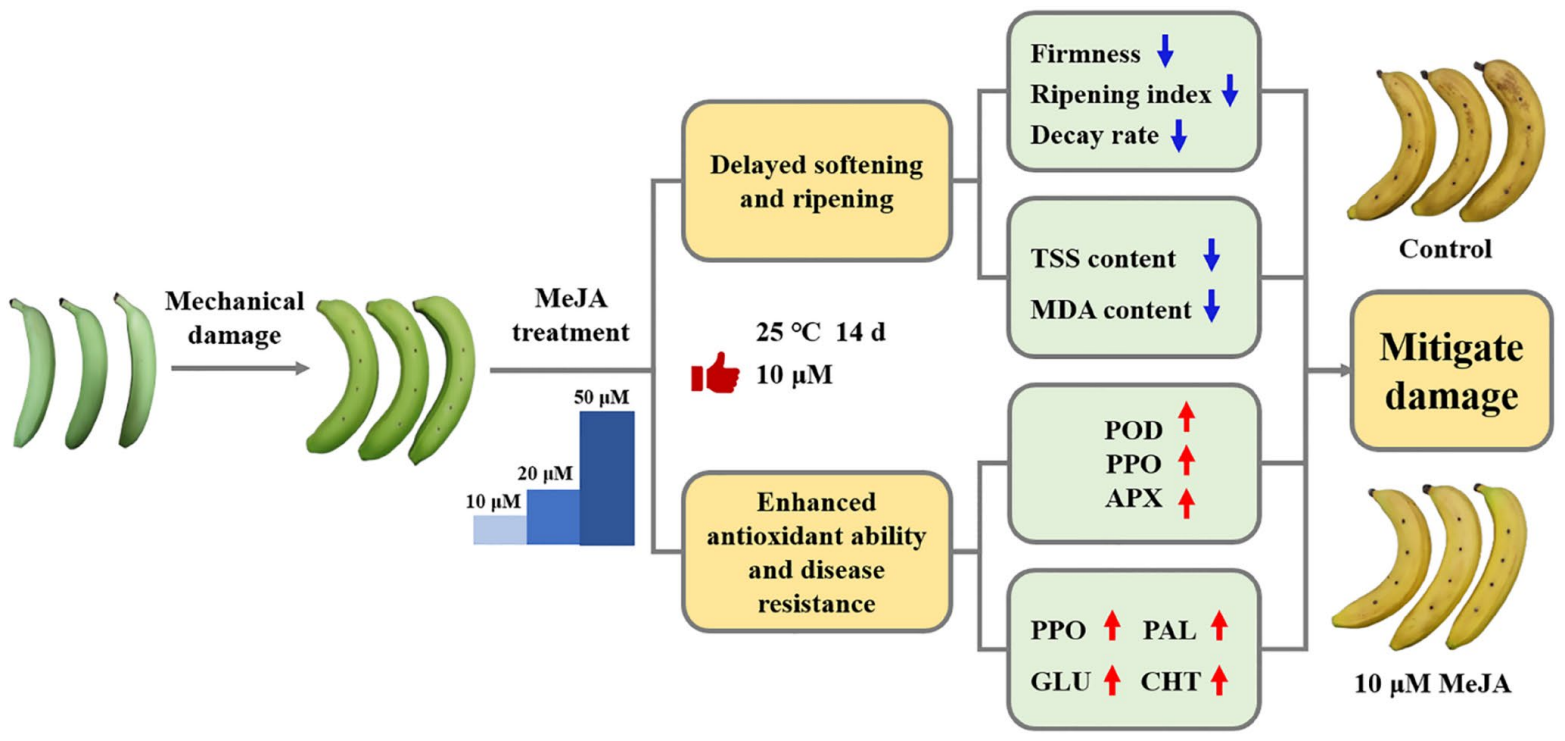
Schematic of the effect of 10 μM MeJA on fruit quality and disease resistance of mechanically damaged banana fruits during storage.

Based on the above results, a proposed model for the regulating mechanism of MeJA treatment in mitigating mechanically injured banana fruit was developed (Figure 6). MeJA treatment maintained fruit firmness, delayed banana softening and yellowing, reduced the rate of respiration intensity and accumulation of MDA in fruit tissues, enhanced antioxidant ability and disease resistance, and finally mitigated mechanically injured banana fruits.

## 5 Conclusion

In this study, the effects of different concentrations of MeJA on mechanically injured banana fruit were evaluated. 10 μM MeJA was the suitable concentration to enhance the scavenging of ROS, reduce membrane lipid peroxidation, strengthen disease resistance, and mitigate the damage caused by mechanical injury to banana fruits. It maintains the quality of the mechanically injured banana fruits and extends their storage period. Our findings provide insight into the postharvest preservation of mechanically injured banana fruits. However, the underlying regulatory mechanisms remain unclear, and further research is needed to explore the changes in expression of genes and metabolites related to the JA signaling pathway and related metabolic pathways after treatment with 10 μM MeJA. So as to reveal the molecular mechanism by which MeJA regulates mechanically injured bananas, and better apply it to alleviate the adverse effects.

## Author Contributions

**Fang Huang:** data curation (equal), formal analysis (equal), investigation (equal), writing – original draft (equal). **Zhenmei Fan:** conceptualization (equal), data curation (equal), formal analysis (equal), writing – original draft (equal). **Ping Yi:** data curation (equal), investigation (equal), writing – review and editing (equal). **Min Huang:** formal analysis (equal), investigation (equal). **Ting Gan:** formal analysis (equal), investigation (equal). **Yuhan Long:** validation (equal), visualization (equal). **Lihong Xie:** conceptualization (equal), methodology (equal), validation (equal), writing – review and editing (equal). **Li Li:** funding acquisition (equal), supervision (equal), validation (equal), writing – review and editing (equal). **Lan Zhang:** investigation (equal). **Hao Dong:** data curation (equal). **Qi Wei:** data curation (equal).

## Funding

This work was supported by the National Key R&D Program of China (2024YFD2100604), Natural Science Foundation of Guangxi (2025GXNSFBA069490), National Natural Science Foundation of China (32272784, 32160732), the Earmarked Fund for CARS‐31, Foundation of Fundamental Research Project from Guangxi Academy of Agricultural Sciences (Gui Nong Ke 2026YT156).

## Conflicts of Interest

The authors declare no conflicts of interest.

## Data Availability

Data are contained within the article.

## References

[fsn371392-bib-0002] Al‐Dairi, M. , P. B. Pathare , R. Al‐Yahyai , H. Jayasuriya , and Z. Al‐Attabi . 2023. “Postharvest Quality, Technologies, and Strategies to Reduce Losses Along the Supply Chain of Banana: A Review.” Trends in Food Science & Technology 134: 177–191. 10.1016/j.tifs.2023.03.003.

[fsn371392-bib-0001] Al‐Dairi, M. , P. B. Pathare , R. Al‐Yahyai , H. Jayasuriya , and Z. Al‐Attabi . 2024. “Evaluation of Chemical Quality Attributes in Bruised Bananas During Storage.” LWT 197: 115904. 10.1016/j.lwt.2024.115904.

[fsn371392-bib-0003] Ali, M. S. , and K.‐H. Baek . 2020. “Jasmonic Acid Signaling Pathway in Response to Abiotic Stresses in Plants.” International Journal of Molecular Sciences 21, no. 2: 621. 10.3390/ijms21020621. 31963549 PMC7013817

[fsn371392-bib-0004] Ba, L. , S. Cao , N. Ji , C. Ma , R. Wang , and D. Luo . 2022. “Exogenous Melatonin Treatment in the Postharvest Storage of Pitaya Fruits Delays Senescence and Regulates Reactive Oxygen Species Metabolism.” Food Science and Technology 42: e15221. 10.1590/fst.15221.

[fsn371392-bib-0005] Chen, H. , X. Lai , L. Wang , et al. 2022. “Ethylene Response Factor MaERF012 Modulates Fruit Ripening by Regulating Chlorophyll Degradation and Softening in Banana.” Food 11, no. 23: 3882. 10.3390/foods11233882. PMC9738063 36496689

[fsn371392-bib-0006] Ding, J. , Y. Hao , B. Liu , Y. Chen , and L. Li . 2023. “Development and Application of Poly (Lactic Acid)/Poly (Butylene Adipate‐Co‐Terephthalate)/Thermoplastic Starch Film Containing Salicylic Acid for Banana Preservation.” Food 12, no. 18: 3397. 10.3390/foods12183397. PMC10529493 37761105

[fsn371392-bib-0007] Fan, Y. , C. Li , J. Zhu , et al. 2022. “Organic Acids Metabolism and GABA Shunt Involved in Maintaining Quality of *Malus domestica* by Methyl Jasmonate Treatment.” Food Research International 160: 111741. 10.1016/j.foodres.2022.111741. 36076423

[fsn371392-bib-0008] Glowacz, M. , N. Roets , and D. Sivakumar . 2017. “Control of Anthracnose Disease via Increased Activity of Defence Related Enzymes in ‘Hass’ Avocado Fruit Treated With Methyl Jasmonate and Methyl Salicylate.” Food Chemistry 234: 163–167. 10.1016/j.foodchem.2017.04.063. 28551220

[fsn371392-bib-0009] Gu, S. , D. Xu , F. Zhou , K. Feng , C. Chen , and A. Jiang . 2022. “Repairing Ability and Mechanism of Methyl Jasmonate and Salicylic Acid on Mechanically Damaged Sweet Cherries.” Scientia Horticulturae 292: 110567. 10.1016/j.scienta.2021.110567.

[fsn371392-bib-0010] Huang, C. , P. Yi , J. Li , et al. 2024. “Exogenous Methyl Jasmonate Alleviates Mechanical Damage in Banana Fruit by Regulating Membrane Lipid Metabolism.” Food 13, no. 19: 3132. 10.3390/foods13193132. PMC11475893 39410165

[fsn371392-bib-0011] Huang, H. , G. Jing , H. Wang , X. Duan , H. Qu , and Y. Jiang . 2014. “The Combined Effects of Phenylurea and Gibberellins on Quality Maintenance and Shelf Life Extension of Banana Fruit During Storage.” Scientia Horticulturae 167: 36–42. 10.1016/j.scienta.2013.12.028.

[fsn371392-bib-0012] Huang, T. , G. Liu , L. Zhu , et al. 2024. “Mitigation of Chilling Injury in Mango Fruit by Methyl Jasmonate Is Associated With Regulation of Antioxidant Capacity and Energy Homeostasis.” Postharvest Biology and Technology 211: 112801. 10.1016/j.postharvbio.2024.112801.

[fsn371392-bib-0013] Ji, N. , J. Wang , Y. Li , M. Li , P. Jin , and Y. Zheng . 2021. “Involvement of PpWRKY70 in the Methyl Jasmonate Primed Disease Resistance Against *Rhizopus stolonifer* of Peaches via Activating Phenylpropanoid Pathway.” Postharvest Biology and Technology 174: 111466. 10.1016/j.postharvbio.2021.111466.

[fsn371392-bib-0014] Ji, N. , J. Wang , X. Zuo , et al. 2021. “PpWRKY45 Is Involved in Methyl Jasmonate Primed Disease Resistance by Enhancing the Expression of Jasmonate Acid Biosynthetic and Pathogenesis‐Related Genes of Peach Fruit.” Postharvest Biology and Technology 172: 111390. 10.1016/j.postharvbio.2020.111390.

[fsn371392-bib-0015] Khaksar, G. , C. Treesubsuntorn , and P. Thiravetyan . 2017. “Effect of Exogenous Methyl Jasmonate on Airborne Benzene Removal by *Zamioculcas zamiifolia*: The Role of Cytochrome P450 Expression, Salicylic Acid, IAA, ROS and Antioxidant Activity.” Environmental and Experimental Botany 138: 130–138. 10.1016/j.envexpbot.2017.03.007.

[fsn371392-bib-0016] Li, J. , M. Azam , A. Noreen , et al. 2023. “Application of Methyl Jasmonate to Papaya Fruit Stored at Lower Temperature Attenuates Chilling Injury and Enhances the Antioxidant System to Maintain Quality.” Food 12, no. 14: 2743. 10.3390/foods12142743. PMC10380080 37509835

[fsn371392-bib-0017] Li, L. , X. He , J. Sun , et al. 2017a. “Cloning, Characterization, and Functional Expression of Phospholipase D *α* cDNA From Banana (*Musa acuminate* L.).” Journal of Food Quality 2017: 1–7. 10.1155/2017/2510949.

[fsn371392-bib-0018] Li, L. , X. He , J. Sun , et al. 2017b. “Responses of Phospholipase D and Antioxidant System to Mechanical Wounding in Postharvest Banana Fruits.” Journal of Food Quality 2017: 1–8. 10.1155/2017/8347306.

[fsn371392-bib-0019] Li, L. , P. Yi , F. Huang , et al. 2022. “Effects of Phospholipase D Inhibitors Treatment on Membrane Lipid Metabolism of Postharvest Banana Fruit in Response to Mechanical Wounding Stress.” Horticulturae 8, no. 10: 901. 10.3390/horticulturae8100901.

[fsn371392-bib-0020] Liao, L. , S. Li , Y. Li , et al. 2022. “Pre‐ or Post‐Harvest Treatment With MeJA Improves Post‐Harvest Storage of Lemon Fruit by Stimulating the Antioxidant System and Alleviating Chilling Injury.” Plants 11, no. 21: 2840. 10.3390/plants11212840. 36365293 PMC9655630

[fsn371392-bib-0021] Liao, Y. , R. Pan , J. Wei , and F. Lv . 2022. “AsJAZ1 Represses the Expression of the Sesquiterpene Synthase Gene Based on the JA Signaling Pathway in *Aquilaria sinensis* (Lour.) Gilg.” Plant Biotechnology Reports 17: 101–109. 10.1007/s11816-022-00758-w.

[fsn371392-bib-0022] Liu, Y. , Y. Liu , Q. Chen , et al. 2023. “Methyl Jasmonate Treatment Alleviates Chilling Injury and Improves Antioxidant System of Okra Pod During Cold Storage.” Food Science & Nutrition 11, no. 4: 2049–2060. 10.1002/fsn3.3241. 37051347 PMC10084972

[fsn371392-bib-0023] Lu, K. , X. Wu , R. Yuan , et al. 2024. “Mechanism of Exogenous Methyl Jasmonate in Regulating the Quality of Fresh‐Cut Chinese Water Chestnuts.” Frontiers in Plant Science 15: 1435066. 10.3389/fpls.2024.1435066. 39220004 PMC11362587

[fsn371392-bib-0024] Maia, V. M. , L. C. C. Salomão , D. L. Siqueira , R. Puschman , V. J. G. Mota Filho , and P. R. Cecon . 2011. “Physical and Metabolic Alterations in ‘Prata Anã’ Banana Induced by Mechanical Damage at Room Temperature.” Scientia Agricola 68, no. 1: 31–36. 10.1590/S0103-90162011000100005.

[fsn371392-bib-0025] Nasrin, T. A. A. , L. Yasmin , M. S. Arfin , et al. 2022. “Preservation of Postharvest Quality of Fresh Cut Cauliflower Through Simple and Easy Packaging Techniques.” Applied Food Research 2, no. 2: 100125. 10.1016/j.afres.2022.100125.

[fsn371392-bib-0026] Pan, L. , X. Chen , W. Xu , et al. 2022. “Methyl Jasmonate Induces Postharvest Disease Resistance to Decay Caused by *Alternaria alternata* in Sweet Cherry Fruit.” Scientia Horticulturae 292: 110624. 10.1016/j.scienta.2021.110624.

[fsn371392-bib-0027] Pan, L. , X. Zhao , M. Chen , Y. Fu , M. Xiang , and J. Chen . 2020. “Effect of Exogenous Methyl Jasmonate Treatment on Disease Resistance of Postharvest Kiwifruit.” Food Chemistry 305: 125483. 10.1016/j.foodchem.2019.125483. 31610420

[fsn371392-bib-0028] Pathare, P. B. , and M. Al‐Dairi . 2022. “Effect of Mechanical Damage on the Quality Characteristics of Banana Fruits During Short‐Term Storage.” Discover Food 2, no. 1: 4. 10.1007/s44187-022-00007-7.

[fsn371392-bib-0029] Peng, Y. , and L. Mao . 2011. “Salicylic Acid, Ethephon, and Methyl Jasmonate Induce the Expression of Phospholipase D in Mechanically‐Wounded Cucumber.” Journal of Horticultural Science and Biotechnology 86, no. 3: 235–240. 10.1080/14620316.2011.11512754.

[fsn371392-bib-0030] Ranjha, M. M. A. N. , S. Irfan , M. Nadeem , and S. Mahmood . 2022. “A Comprehensive Review on Nutritional Value, Medicinal Uses, and Processing of Banana.” Food Reviews International 38, no. 2: 199–225. 10.1080/87559129.2020.1725890.

[fsn371392-bib-0031] Shinga, M. H. , and O. A. Fawole . 2023. “ *Opuntia ficus indica* Mucilage Coatings Regulate Cell Wall Softening Enzymes and Delay the Ripening of Banana Fruit Stored at Retail Conditions.” International Journal of Biological Macromolecules 245: 125550. 10.1016/j.ijbiomac.2023.125550. 37356689

[fsn371392-bib-0032] Song, Y. , C. Hu , Y. Xue , J. Gu , J. He , and Y. Ren . 2022. “24‐Epibrassinolide Enhances Mango Resistance to *Colletotrichum gloeosporioides* via Activating Multiple Defense Response.” Scientia Horticulturae 303: 111249. 10.1016/j.scienta.2022.111249.

[fsn371392-bib-0033] Wang, H. , X. Kou , C. Wu , G. Fan , and T. Li . 2020. “Methyl Jasmonate Induces the Resistance of Postharvest Blueberry to Gray Mold Caused by *Botrytis cinerea* .” Journal of the Science of Food and Agriculture 100, no. 11: 4272–4281. 10.1002/jsfa.10469. 32378217

[fsn371392-bib-0034] Wang, Y. , Z. Luo , and R. Du . 2015. “Nitric Oxide Delays Chlorophyll Degradation and Enhances Antioxidant Activity in Banana Fruits After Cold Storage.” Acta Physiologiae Plantarum 37, no. 4: 74. 10.1007/s11738-015-1821-z.

[fsn371392-bib-0035] Wantat, A. , K. Seraypheap , and P. Rojsitthisak . 2022. “Effect of Chitosan Coatings Supplemented With Chitosan‐Montmorillonite Nanocomposites on Postharvest Quality of ‘Hom Thong’ Banana Fruit.” Food Chemistry 374: 131731. 10.1016/j.foodchem.2021.131731. 34896958

[fsn371392-bib-0036] Xu, D. , J. Zuo , P. Li , et al. 2020. “Effect of Methyl Jasmonate on the Quality of Harvested Broccoli After Simulated Transport.” Food Chemistry 319: 126561. 10.1016/j.foodchem.2020.126561. 32172047

[fsn371392-bib-0037] Xu, F. , Y. Liu , J. Xu , and L. Fu . 2019. “Influence of 1‐Methylcyclopropene (1‐MCP) Combined With Salicylic Acid (SA) Treatment on the Postharvest Physiology and Quality of Bananas.” Journal of Food Processing and Preservation 43, no. 3: e13880. 10.1111/jfpp.13880.

[fsn371392-bib-0038] Yang, Z. , S. Cao , X. Su , and Y. Jiang . 2014. “Respiratory Activity and Mitochondrial Membrane Associated With Fruit Senescence in Postharvest Peaches in Response to UV‐C Treatment.” Food Chemistry 161: 16–21. 10.1016/j.foodchem.2014.03.120. 24837916

[fsn371392-bib-0039] Yi, P. , L. Li , J. Sun , et al. 2022. “Characterization and Expression of Phospholipase D Putatively Involved in *Colletotrichum musae* Disease Development of Postharvest Banana Fruit.” Horticulturae 8, no. 4: 312. 10.3390/horticulturae8040312.

[fsn371392-bib-0040] Yu, K. , J. Xu , L. Zhou , L. Zou , and W. Liu . 2021. “Effect of Chitosan Coatings With Cinnamon Essential Oil on Postharvest Quality of Mangoes.” Food 10, no. 12: 3003. 10.3390/foods10123003. PMC8700884 34945553

[fsn371392-bib-0041] Yuan, S. , H. Jiang , Y. Wang , et al. 2024. “A 3R‐MYB Transcription Factor Is Involved in Methyl Jasmonate‐Induced Disease Resistance in *Agaricus bisporus* and Has Implications for Disease Resistance in *Arabidopsis* .” Journal of Advanced Research 73: 117–131. 10.1016/j.jare.2024.08.037. 39233001 PMC12225956

[fsn371392-bib-0042] Yun, Z. , H. Gao , X. Chen , X. Duan , and Y. Jiang . 2022. “The Role of Hydrogen Water in Delaying Ripening of Banana Fruit During Postharvest Storage.” Food Chemistry 373: 131590. 10.1016/j.foodchem.2021.131590. 34802805

[fsn371392-bib-0043] Zhang, J. , X. Chen , Q. Liu , et al. 2024. “Slightly Acidic Electrolyzed Water Treatment Enhances the Quality Attributes and the Storability of Postharvest Litchis Through Regulating the Metabolism of Reactive Oxygen Species.” Food Chemistry: X 23: 101644. 10.1016/j.fochx.2024.101644. 39148531 PMC11325003

[fsn371392-bib-0044] Zhang, X. , D. Min , F. Li , N. Ji , D. Meng , and L. Li . 2017. “Synergistic Effects of l‐Arginine and Methyl Salicylate on Alleviating Postharvest Disease Caused by *Botrysis Cinerea* in Tomato Fruit.” Journal of Agricultural and Food Chemistry 65, no. 24: 4890–4896. 10.1021/acs.jafc.7b00395. 28535671

[fsn371392-bib-0045] Zhao, L. , F. He , B. Li , et al. 2022. “Transcriptomic Analysis of the Mechanisms Involved in Enhanced Antagonistic Efficacy of *Meyerozyma guilliermondii* by Methyl Jasmonate and Disease Resistance of Postharvest Apples.” LWT 160: 113323. 10.1016/j.lwt.2022.113323.

[fsn371392-bib-0046] Zhou, X. , J. Cheng , J. Sun , et al. 2023. “Effect of Red Visible Lighting on Postharvest Ripening of Bananas via the Regulation of Energy Metabolism.” Horticulturae 9, no. 7: 840. 10.3390/horticulturae9070840.

[fsn371392-bib-0047] Zhu, L. , H. Yu , X. Dai , M. Yu , and Z. Yu . 2022. “Effect of Methyl Jasmonate on the Quality and Antioxidant Capacity by Modulating Ascorbate‐Glutathione Cycle in Peach Fruit.” Scientia Horticulturae 303: 111216. 10.1016/j.scienta.2022.111216.

[fsn371392-bib-0048] Zhu, S. , and B. Ma . 2007. “Benzothiadiazole‐ or Methyl Jasmonate‐Induced Resistance to *Colletotrichum musae* in Harvested Banana Fruit Is Related to Elevated Defense Enzyme Activities.” Journal of Horticultural Science and Biotechnology 82, no. 4: 500–506. 10.1080/14620316.2007.11512265.

[fsn371392-bib-0049] Zhu, X. , L. Jiang , Y. P. Cai , and Y. P. Cao . 2021. “Functional Analysis of Four Class III Peroxidases From Chinese Pear Fruit: A Critical Role in Lignin Polymerization.” Physiology and Molecular Biology of Plants 27, no. 3: 515–522. 10.1007/s12298-021-00949-9. 33854280 PMC7981345

[fsn371392-bib-0050] Zhu, X. , Z. Song , Q. Li , J. Li , W. Chen , and X. Li . 2020. “Physiological and Transcriptomic Analysis Reveals the Roles of 1‐MCP in the Ripening and Fruit Aroma Quality of Banana Fruit (Fenjiao).” Food Research International 130: 108968. 10.1016/j.foodres.2019.108968. 32156402

